# T-cell lymphoma with a granulomatous lesion of the lungs after autologous hematopoietic stem cell transplantation for Epstein–Barr virus-positive diffuse large B-cell lymphoma: a unique rare case of metachronous B-cell and T-cell lymphoma

**DOI:** 10.1186/s13000-020-01038-3

**Published:** 2020-10-09

**Authors:** Yusuke Kajimoto, Yasuhiro Terasaki, Mika Terasaki, Shinobu Kunugi, Yugo Okabe, Satoshi Wakita, Koiti Inokuchi, Akira Shimizu

**Affiliations:** 1 Department of Analytic Human Pathology, Nippon Medical School, 1-25-16, Nezu, Bunkyo-ku, Tokyo, 113-0031 Japan; 2 Division of Pathology, Nippon Medical School Hospital, 1-1-5, Sendagi, Bunkyo-ku, Tokyo, 113-8602 Japan; 3 Department of Hematology, Nippon Medical School Hospital, 1-1-5, Sendagi, Bunkyo-ku, Tokyo, 113-8602 Japan

**Keywords:** Epstein–Barr virus, Primary immunodeficiency disorder, Common variable immunodeficiency, Peripheral T-cell lymphoma, Diffuse large B-cell lymphoma, Post-transplant lymphoproliferative disorder, Autologous hematopoietic stem cell transplantation, Granuloma

## Abstract

**Background:**

Epstein–Barr virus (EBV) is associated with the pathogenesis of a variety of malignancies, most notably lymphomas. Especially in the background of immunodeficiency, such as primary immunodeficiency disorder (PID) and post-transplant lymphoproliferative disorder (PTLD), the role of EBV might be crucial. PIDs are rare heterogeneous diseases affecting the development and/or the function of the innate and adaptive immune system. Malignancy is the second-highest cause of death after infection, and lymphoma accounts for about half of malignancies. The most frequently reported lymphoma type is diffuse large B-cell lymphoma (DLBCL) and the incidence of T-cell lymphoma is rare. PTLDs are also rare serious lymphoid and/or plasmacytic proliferative disorders that occur after undergoing solid organ or hematopoietic stem cell transplantation (HSCT). In the context of HSCT, most reported PTLDs have occurred in patients who received allogenic HSCT, but only a few cases have been reported in autologous HSCT (AutoHSCT) recipients.

**Case presentation:**

A 53-year-old female patient initially presented with enlargement of the left cervical lymph nodes and was diagnosed with EBV-positive DLBCL. She was treated with R-CHOP, R-ACES, and AutoHSCT and went into remission. Four years later, computed tomography results revealed multiple lung nodules and abnormal infiltration, and sustained and progressing hypogammaglobulinemia was observed. The pathological specimen of video-assisted thoracoscopic surgical lung biopsy demonstrated extensive invasion of lymphocytes with notable granuloma findings. Flow cytometric immunophenotyping analysis showed that lymphocytes were positive for CD3 and CD5; especially, CD3 was expressed in the cytoplasm. Southern blot analysis revealed rearrangements of the T-cell receptor Cβ1 gene. She was diagnosed with peripheral T-cell lymphoma, not otherwise specified, accompanied by notable granulomatous lesions.

**Conclusion:**

Here, as a unique case of metachronous B-cell and T-cell lymphoma, we report a rare case of T-cell lymphoma that mainly affected the lungs with the presentation of notable granulomatous findings following AutoHSCT for EBV-positive DLBCL at the age of 53 years. These lung lesions of granulomatous T-cell lymphoma could be related to the underlying primary immunodeficiency background associated with sustained hypogammaglobulinemia.

## Background

Epstein–Barr virus (EBV) is associated with the pathogenesis of a variety of malignancies, most notably lymphomas. Especially in the background of immunodeficiency, such as primary immunodeficiency disorder (PID) and post-transplant lymphoproliferative disorder (PTLD), the role of EBV might be crucial [1]. PIDs are heterogeneous diseases affecting the development and/or the function of various components of the innate and adaptive immune system [2]. The prevalence of PIDs is approximately 41–51:100,000. Malignancy is the second-highest cause of death after infection, and lymphoma accounts for approximately half of malignancies in both children and adults [3, 4]. The risk of lymphoma increases up to 10 times in PID patients, and EBV is associated with 30–60% of lymphoma cases in PIDs [4, 5]. In PIDs, the most frequently reported lymphoma types are diffuse large B-cell lymphoma (DLBCL; 33.5%) and Burkitt’s lymphoma (7.1%), but the incidence of T-cell lymphoma is rare [1, 6, 7].

PTLDs are also rare serious lymphoid and/or plasmacytic proliferative disorders that occur after solid organ or hematopoietic stem cell transplantation (HSCT) [8]. The incidence of PTLDs is 30–50 times higher than that in the general population and ranges from 2 to 10%, whereas the incidence of PTLDs after HSCT is less than 1% [9, 10]. Most PTLDs are of B-cell origin and are related to EBV infection. T-cell PTLDs, in contrast, constitute fewer than 15% of PTLDs in Western countries [11]. In the context of HSCT, most reported PTLDs have occurred in patients who received allogenic HSCT (AlloHSCT). However, only 25 published cases of PTLD following autologous HSCT (AutoHSCT) have been reported as case reports, with six cases having a T-cell origin reported (Table 1) [12–32]. Here, as a unique case of metachronous B-cell and T-cell lymphoma, we report a rare case of T-cell lymphoma that mainly affected the lungs with notable granulomatous findings following AutoHSCT with sustained hypogammaglobulinemia against EBV-positive DLBCL at the age of 53 years.

**Table 1:** Summary of the clinicopathologic features of T-cell lymphoproliferative disorder following autologous hematopoietic stem cell transplantation

	Age at onset of first neoplasm	Histological diagnosis of first neoplasm	Therapy before AutoHSCT	Histological diagnosis of T-cell LPD	Histological findings	EBV (ISH or PCR)	Areas of T-cell LPD	Time of T-cell LPD onset after HSCT	Outcome	Reference
1	48 years	HL	(1) Doxorubicin-based chemotherapy (2) High-dose cytosine arabinoside (3) Conditioning regimen that included cyclophosphamide, etoposide, and ranimustine	T-cell PTLD	(Lymph node) Lymphoid hyperplasia with mild sinus histiocytosis, as well as proliferation of epithelioid cells.	Positive	Mesenteric lymph nodes	2 years and 6 months	Fatal	[12]
2	61 years	AITL	(1) Four cycles of CHOP (2) High-dose etoposide (3) Two cycles of CHOP (4) Ranimustine, cyclophosphamide, etoposide, and carboplatin	T-LGL	(Blood) Pronounced lymphocytosis of large-sized lymphoid cells with round to indented nuclei, coarse chromatin, and azurophilic cytoplasmic granules.	Negative	Bone marrow	1 month	Survival	[13]
3	62 years	FL	(1) Eight cycles of CHOP (2) Two cycles of MINE (3) Etoposide (4) Ranimustine, carboplatin, etoposide, and cyclophosphamide	AITL	(Lymph node) Diffuse infiltration of medium to large-sized lymphoid cells with predominant proliferation of small blood vessels.	Positive	Cervical and supraclavicular lymph nodes	4 months	Resolved	[14]
4	49 years	AITL	(1) Five cycles of CHOP (2) Salvage chemotherapy: ESHAP (3) MCVAC	T-cell PTLD	(Autopsy) Lymphoid cells in the bone marrow, portal area of the liver, lymph nodes and lungs. The hypocellularity in the marrow with the proliferation of macrophages and marked hemophagocytosis. Macrophages in the marrow, liver, lymph nodes, and lungs.	Positive	Bone marrow, liver, lungs, lymph nodes	3 months	Fatal	[15]
5	47 years	DLBCL	High-dose radiotherapy and chemotherapy	Enteropathy type T-cell lymphoma	(Jejunum) An atypical destructive lymphoid infiltration with intraepithelial lymphocytes in the superficial mucosa and in the glands.	Negative	Jejunum, duodenum, mesenteric lymph nodes	6 years and 3 months	Resolved	[16]
6	53 years	MM	(1) Three cycles of VAD (2) High-dose melphalan: Prednisolone and cyclosporin A for erythroderma	AITL	(Lymph node) Nodular infiltration of atypical lymphocytes with large nucleoli.	Positive	Brain, axillary and inguinal lymph nodes	10 months	Resolved	[17]
Our case	53 years	DLBCL	(1) Six cycle of R-CHOP (2) Three cycles of R-ACES	PTCL-NOS	(Lung) Diffuse infiltration of small to medium-sized lymphocytes with multiple granulomas.	Positive	Supraclavicular, mediastinal, hilar, paraaortic, mesenteric lymph nodes, lungs	4 years	Fatal	

*HL* Hodgkin lymphoma, *AITL* angioimmunoblastic T-cell lymphoma, *FL* follicular lymphoma, *DLBCL* diffuse large B-cell lymphoma, *MM* multiple myeloma, *PTCL-NOS* peripheral T-cell lymphoma, not otherwise specified, *T-LGL* T-cell large granular lymphocytic leukemia, *R-CHOP* rituximab, cyclophosphamide, doxorubicin, and vincristine, *MINE* mesna, ifosfamide, mitoxantrone, and etoposide, *ESHAP* etoposide, cytarabine, cisplatin, and methylprednisolone, *MCVAC* ranimustine, cytarabine, etoposide, and cyclophosphamide, *VAD* vincristine, doxorubicin, and dexamethasone, *CVP* cyclophosphamide, prednisolone, and vincristine, *AutoHASCT* autologous hematopoietic stem cell transplantation, *R-ACES* rituximab, high-dose Ara C, carboplatin, etoposide, and steroids

## Case presentation

### First lymphoid neoplasm

**Fig. 1:**
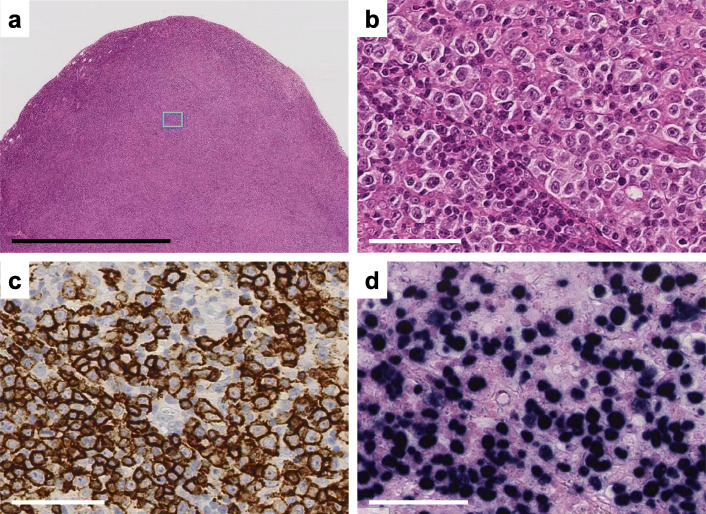
Diffuse large B-cell lymphoma in the cervical lymph node. **a** The node architecture was effaced by diffuse infiltration lymphocytes. **b** Higher magnification of the boxed area in (**a**) reveals diffuse infiltration of the medium and large lymphocytes. **c** Immunostaining revealed that the lymphocytes were positive for CD20. **d** In situ hybridization showed that these cells were positive for EBV-encoded small RNA (EBER). **b-d** Serial sections. Scale bar: 2 mm (**a**), 60 μm (**b-d**)

A 53-year-old woman initially presented with enlargement of the left cervical lymph nodes (LNs) in 2013. Cervical node biopsy revealed the diffuse infiltration of atypical medium to large lymphocytes (Fig. 1a, b). Laboratory studies showed low IgG and IgA levels (445 and 83 mg/dL, respectively) with normal IgM levels. Immunohistochemistry analysis showed that lymphocytes were positive for CD20 (Fig. 1c) and negative for CD3 and CD10 (data not shown). Furthermore, in situ hybridization (ISH) revealed that lymphocytes were positive for EBV-encoded small RNA (EBER) (Fig. 1d). She was diagnosed with EBV-positive DLBCL. She received six cycles of R-CHOP (rituximab, cyclophosphamide, doxorubicin, vincristine, and prednisone) chemotherapy in 2013 and was treated with three cycles of R-ACES (rituximab, high-dose Ara C, carboplatin, etoposide, and steroids) chemotherapy followed by AutoHSCT in 2014. She then achieved complete remission.

### Second lymphoid neoplasm

**Fig. 2:**
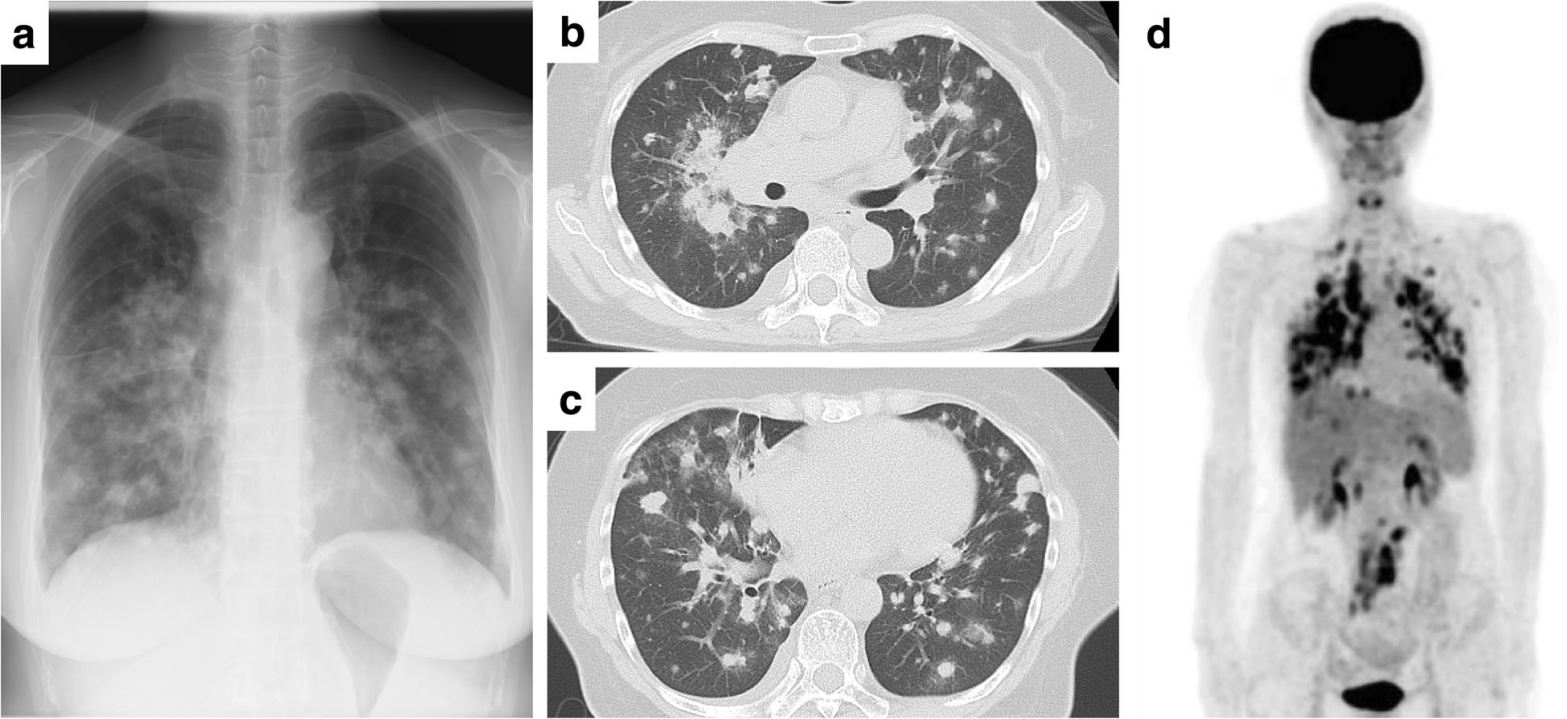
Radiographic features of T-cell lymphoma. **a-c** Chest X-ray and a computed tomography scan showed multifocal pulmonary consolidations and nodules. **d** 18F-fluorodeoxyglucose positron emission tomography (FDG-PET) scan showed the accumulation of FDG in the bilateral lung nodules and the lymph nodes

She was temporarily affected by pneumonia in 2017 and paranasal sinusitis in 2018. Subsequently, she presented with wheezing, and chest X-ray and computed tomography revealed multiple lung nodules and consolidations (Fig. 2a-c) in 2018. Positron emission tomography showed the abnormal accumulation of 18F-fluorodeoxyglucose (FDG) in bilateral lungs (Fig. 2d). In addition, the abnormal uptake of FDG was observed in the supraclavicular, mediastinal, hilar, paraaortic, and mesenteric LNs (Fig*.* 2d).

**Fig. 3:**
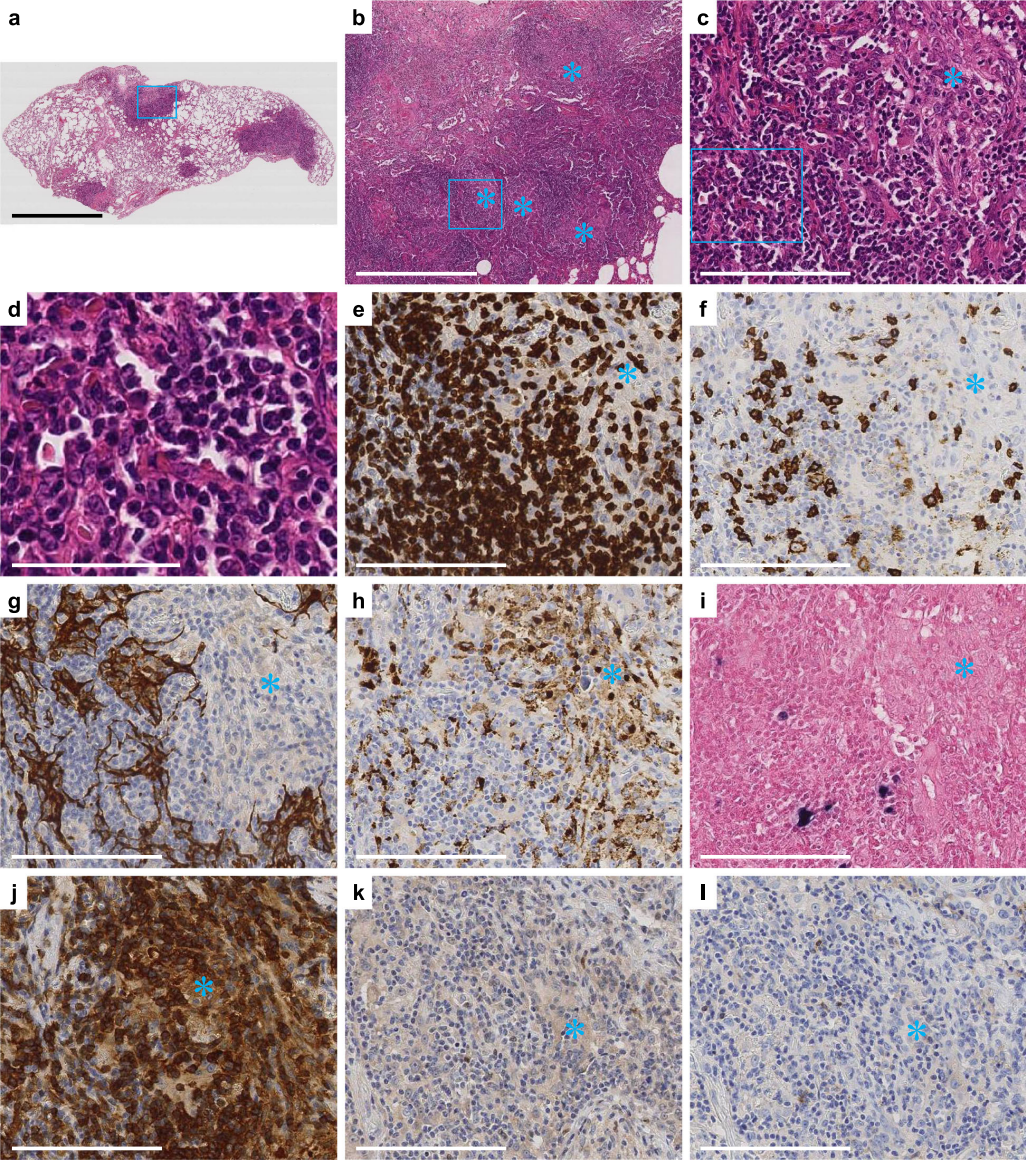
Pathologic findings of the video-assisted thoracoscopic surgery (VATS) specimen. **a** In the lung tissue, multiple nodules were observed. **b**, **c** High power views showing notable infiltration of lymphocytes with multiple granulomas (*). **d** The lymphocytes were small to medium-sized with slight nuclear irregularities. **b-d** Higher magnification of the boxed area in (**a**), (**b**), and (**c**), respectively. **e**, **f** The lymphocytes were positive for CD3 (**e**) and focally positive for CD20 (**f**). **g** The lymphocytes destructively infiltrated the alveolar epithelium (AE1/AE3). **h** Epithelioid cells of the granulomas were positive for CD68. **i** Some of the lymphocytes were positive for EBER. **j-l** The lymphocytes were positive for CD4 (**j**) and negative for BCL-6 (**k**) and CD10 (**l**). **c**, **e-l** Serial sections. Scale bar: 7 mm (**a**), 1 mm (**b**), 150 μm (**c**, **e-l**), 60 μm (**d**)

Laboratory tests showed the gradual progression of hypogammaglobulinemia with low IgG, IgA, and IgM levels (367, 25, and 33 mg/dL, respectively). Polymerase chain reaction was conducted on the whole-blood EBV DNA, and the DNA levels were found to have increased up to 1.0 × 10^3^ copies/mL from 2.0 × 10^2^ copies/mL. Video-assisted thoracoscopic surgery (VATS) was performed for a biopsy of the left upper lobe of the lung. Macroscopic examination of the lung revealed multiple nodules, whereas the microscopic examination demonstrated diffuse infiltration of lymphocytes with multiple granulomas (Fig. 3a-c). The lymphocytes were mainly small to medium-sized and contained slight nuclear irregularities (Fig. 3d). Immunohistochemical staining revealed that lymphocytes were positive for CD3 and focally positive for CD20 (Fig. 3e, f). The lymphocytes had destructively infiltrated the alveolar epithelium based on immunostaining for AE1/AE3 (Fig. 3g). The lymphocytes were positive for CD4 (Fig. 3j) and focally positive for CD8, TIA1, and granzyme B (data not shown). The lymphocytes were negative for BCL-6, CD10 (Fig. 3k, l), CD30, CD56, and ALK (data not shown). In the granulomas, epithelioid cells were positive for CD68 (Fig. 3h). ISH results revealed that some lymphocytes were positive for EBER (Fig. 3i). Grocott staining, Wade-Fite staining, and Giemsa staining were negative. The blood test and lung pathological findings did not show any evidence of infection or other granulomatous lesions such as granulomatous angiitis and sarcoidosis.

**Fig. 4:**
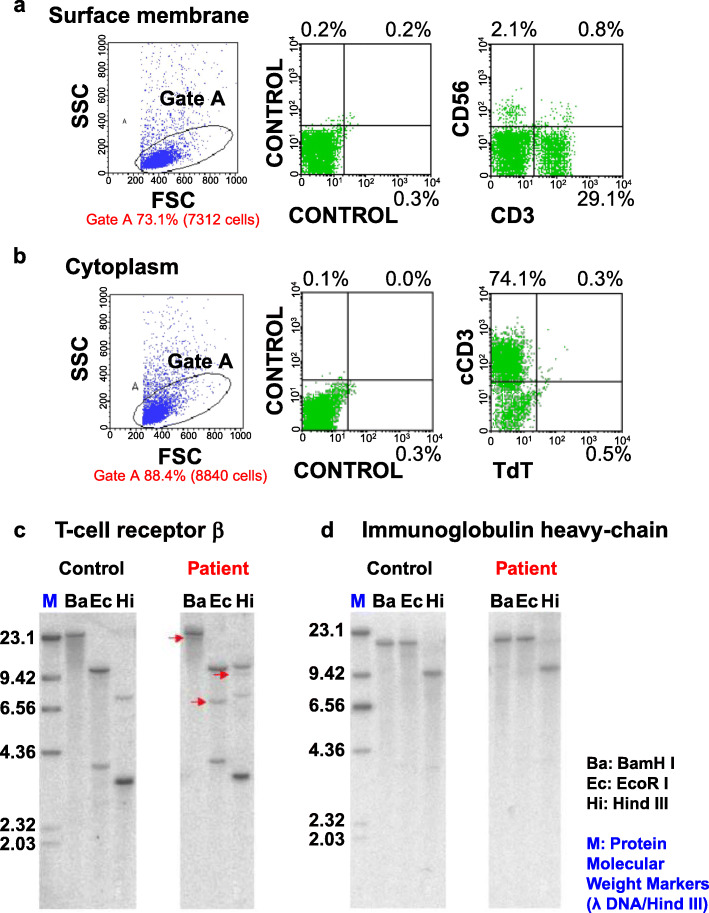
Flow cytometric and Southern blot analysis of gene rearrangements in video-assisted thoracoscopic surgery (VATS) specimen. **a**, **b** CD3 was focally expressed on the surface membrane (**a**) and expressed in the cytoplasm (**b**). **c**, **d** DNA from the lung tissue involved in lymphocyte infiltration after autologous hematopoietic stem cell transplantation (AutoHSCT) was digested with selected restriction enzymes (BamHI (Ba), EcoRI (Ec), and Hind III (Hi) for the T-cell receptor β (TCR β) gene and immunoglobulin heavy chain (IgH) gene). Control DNA was obtained from donated cord blood. DNA was probed with either a Cβ1 probe or a JH probe. TCR β gene rearrangements (arrows) were detected upon Ba, Ec, and Hi digestion (**c**). No rearrangement of IgH gene was found (**d**)

Flow cytometric immunophenotyping analysis of the specimen revealed that the proportion of surface CD3 was 29.9% and that of cytoplasmic CD3 was 74.4% (Fig. 4a, b). The proportions of CD2+, CD4+, CD5+, CD7+, and CD8+ cells were 92.5, 73.6, 84.7, 71.0, and 16.0%, respectively. In contrast, the proportions of cells positive for CD10, CD19, CD20, CD25, surface Ig, and TdT were all < 10% (data not shown). Southern blot analysis of the specimen indicated rearrangements of the T-cell receptor Cβ1 gene, and rearrangements of the immunoglobulin heavy chain gene were not found (Fig. 4c, d). She was subsequently diagnosed with peripheral T-cell lymphoma, not otherwise specified (PTCL-NOS), accompanied by notable granulomatous lesions.

The patient was treated with prednisolone and received AlloHSCT, which was followed by tacrolimus and methotrexate. However, she did not achieve neutrophil engraftment and developed pneumonia. Finally, she died of septic shock on day 20 post-AlloHSCT approximately 8 months after the diagnosis of T-cell lymphoma.

## Discussion

We describe a case of T-cell lymphoma after chemotherapy and AutoHSCT for EBV-positive DLBCL as a unique rare case of metachronous B-cell and T-cell lymphoma. Our patient was diagnosed with EBV-positive DLBCL of the cervical LNs at the age of 53 years, and multiple lung lesions of PTCL-NOS associated notable granulomas were observed 4 years after complete remission following chemotherapy and AutoHSCT for DLBCL with sustained hypogammaglobulinemia.

EBV is etiologically linked to a remarkably wide range of lymphoproliferative lesions, including malignant lymphomas, as EBV-associated lymphoproliferative disorders (LPDs) [33]. It was recently reported that EBV-associated LPDs could be categorized into two groups, including disorders in which the host is usually immunocompetent and the role of EBV might be secondary or essential in only a subset of patients such as DLBCL, and disorders that arise in patients with immunodeficiency in which the role of EBV might be crucial, such as LPD associated with PID or PTLD, etc. There is a strong association between the pattern of EBV latency and the immune status of the host, suggesting that the underlying immune condition and the microenvironment are essential for the pathogenesis and manifestations of lymphomas associated with EBV [1].

Our patient developed EBV-positive DLBCL at the age of 53 years. EBV-positive DLBCL usually develops in individuals aged > 50 years, with a peak in the eighth decade [34], thus the onset age of DLBCL of our case seemed relatively young. Our patient also showed sustained and progressive hypogammaglobulinemia with recurrent respiratory infections after R-CHOP for DLBCL. Because hypogammaglobulinemia has been recognized before AutoHSCT for DLBCL treatment, this condition could not be related to transplantation. Thus, the age of onset for EBV-positive DLBCL, hypogammaglobulinemia, and respiratory infections, which comprised the background of our case, led us to consider the possibility of underlying primary immunodeficiency, such as PID including common variable immunodeficiency (CVID).

Monogenic and other genetic defects of the immune system are categorized as PIDs, which affect various components of the immune system with susceptibility to infections but also to malignancies, including lymphoma [2, 6, 35, 36]. Recent investigations reported that the risk of lymphoma is increased tenfold in PID patients and that EBV is associated with 30–60% of lymphoma cases in PIDs [4, 5]. CVID is one of the most prevalent types of PIDs, occurring in approximately 1:50,000–1:25,000 individuals, and is typically characterized by significantly decreased serum levels of IgG with low IgA and/or IgM and recurrent bacterial infections [37–39]. CVID is most often diagnosed in individuals between the ages of 20 and 40 years, however, can occur at any age [37, 39].

In our case, there was indeed an EBV-positive DLBCL first occurring at the age of 53 years, with both IgG and IgA hypogammaglobulinemia at the initial diagnosis of DLBCL, as well as marked hypogammaglobulinemia that persisted and progressed for at least 4 years after DLBCL treatment until the onset of T-cell lymphoma with prominent granulomatous lesions. Therefore, a potential immune deficiency, such as CVID, cannot be ruled out in our case as one of the causes of this unique duplex T-cell after B-cell lymphoma.

In our case, metachronous tumor of T-cell lymphoma occurred and mainly affected the lungs 4 years after AutoHSCT for EBV-positive DLBCL, and thus, our case could be regarded as T-cell PTLD. PTLDs are lymphoid and/or plasmacytic proliferation disorders, including lymphoma, which develop as a consequence of immunosuppression after transplantation [8, 40]. In the context of HSCT, most cases of PTLD are reported in patients who received AlloHSCT and the incidence of PTLDs after AlloHSCT is less than 1% [9, 10]. Moreover, to the best of our knowledge, only 25 published cases of PTLD following AutoHSCT have been reported as case reports, with six cases of T-cell origin after AutoHSCT [12–32]. Thus our case could be regarded as a very rare case of T-cell PTLD after AutoHSCT. We reviewed the clinicopathological data of seven previously reported cases of T-cell PTLD after AutoHSCT including this case and found that five of seven (71.4%) cases were EBV infection-positive and two of seven (28.6%) cases had macrophage proliferative lesions (Table 1). Therefore, although EBV-positive findings in our case were considered relatively low, the possibility that EBV infection has some influence on the development of T-cell lymphoma in our case cannot be completely ruled out, in addition to the possibility that background immunodeficiency might have been involved. However, the most recent WHO classification of lymphoid neoplasms favors these rarely reported lesions of PTLD after AutoHSCT as being more likely iatrogenic and related to the therapy than related to the transplant itself [8]. Thus, we can use the term “PTLD-like lesion” for the T-cell lymphoma lesion in our case, if we use the term “PTLD”.

In our case, the VATS specimen showed notable lymphocyte infiltration and multiple granulomas. No clinicopathological cause of the granuloma was found such as tuberculosis, fungal infection, sarcoidosis, or granulomatous angiitis. As a differential diagnosis, the granulomatous findings can be cited as being related to Lennert’s lymphoma (LeL), a rare variant of PTCL-NOS, characterized by prominent small clusters of epithelioid histiocytes [41, 42]. However, LeL was reported to comprise only 0.71% of PTCLs and relatively rarely invades the extranodal foci; therefore, this pulmonary lesion is not considered a typical LeL-related finding [43, 44]. In contrast, considering hypogammaglobulinemia in our patient, these granulomatous findings could also be interpreted as histological changes associated with the underlying immunodeficiency of this case, as mentioned previously, in addition to a PTCL-NOS lesion. This is because recent studies reported that 1–4% of PID patients have granulomas and the most common types of granulomas are sino-pulmonary (50%), hematologic-lymphoid (33%), and skin (16%); further, 42% are considered to have prominent granulomas [45].

In conclusion, we report a rare case of T-cell lymphoma mainly affecting the lungs with notable granulomatous findings that developed post-AutoHSCT for EBV-positive DLBCL as a unique case of metachronous B-cell and T-cell lymphoma. These lung lesions of granulomatous T-cell lymphoma could be related to an underlying primary immunodeficiency background associated with sustained hypogammaglobulinemia.

## Data Availability

The data and materials are available upon request from the corresponding author.

## Abbreviations

PID: Primary immunodeficiency disorder
CVID: Common variable immunodeficiency
DLBCL: Diffuse large B-cell lymphoma
EBER: EBV-encoded small RNA
EBV: Epstein–Barr virus
HSCT: Hematopoietic stem cell transplantation
PTCL-NOS: peripheral T-cell lymphoma, not otherwise specified
PTLD: Post-transplant lymphoproliferative disorder
VATS: Video-assisted thoracoscopic surgery
